# Effects of two pre-workout supplements on concentric and eccentric force production during lower body resistance exercise in males and females: a counterbalanced, double-blind, placebo-controlled trial

**DOI:** 10.1186/s12970-017-0203-x

**Published:** 2017-11-28

**Authors:** Grant M. Tinsley, Matthew A. Hamm, Amy K. Hurtado, Austin G. Cross, Jose G. Pineda, Austin Y. Martin, Victor A. Uribe, Ty B. Palmer

**Affiliations:** Energy Balance & Body Composition Laboratory, Musculoskeletal Assessment Laboratory, Department of Kinesiology & Sport Management, Texas Tech University, Box 43011, Lubbock, TX 79409 USA

**Keywords:** Caffeine, Citrulline malate, BCAA, Creatine

## Abstract

**Background:**

Pre-workout supplements purportedly enhance feelings of energy, reduce fatigue and improve exercise performance. The purpose of this study was to examine the performance effects of caffeinated and non-caffeinated multi-ingredient pre-workout supplements.

**Methods:**

In a counterbalanced, double-blind, placebo-controlled design, eccentric and concentric force production during lower body resistance exercise on a mechanized squat device were assessed after supplement ingestion. Repetitions-in-reserve/RPE and subjective feelings of energy, focus and fatigue were also examined. Twenty-one resistance-trained adults (12 F, 9 M) completed three conditions in random order: caffeinated supplement, non-caffeinated supplement and placebo. Subjects were not informed of the presence of a placebo condition. Thirty minutes after supplement ingestion, a 3-repetition maximum test and 5 sets of 6 repetitions were completed using the squat device. Each repetition involved 4-s eccentric and concentric phases, and the force signal throughout each repetition was sampled from a load cell contained within the squat device. The scaled and filtered force signals were analyzed using customized software. Repeated measures analysis of variance and appropriate follow-up analyses were utilized to compare dependent variables, and relevant effect sizes (d) were calculated.

**Results:**

Supplement or placebo ingestion led to similar subjective responses (*p* > 0.05). Energy (+8 to 44%; d = 0.3 to 0.8) and focus (+8 to 25%; d = 0.3 to 0.5) were acutely increased by supplement or placebo ingestion and decreased as the exercise session progressed. Fatigue was acutely decreased by supplement or placebo ingestion (−7 to 38%; d = −0.1 to −0.6) and increased as the exercise session progressed. Eccentric and concentric forces were unimproved by supplementation during the exercise sets for both sexes. In the non-caffeinated supplement condition only, maximal eccentric force production was lower during sets 3 to 5, as compared to set 1 (*p* < 0.05). Effect size data indicated that both the caffeinated and non-caffeinated supplements may contribute to small increases in concentric force production in males (+5 to 20%, d = 0.2 to 0.4 relative to placebo), but not females.

**Conclusions:**

As compared to placebo, caffeinated and non-caffeinated multi-ingredient pre-workout supplements failed to improve concentric and eccentric force production. In males, effect size data indicate a possible small benefit of supplementation on concentric force production, although this was not statistically significant. When resistance-trained subjects were unaware of the presence of a placebo, resistance exercise performance was similar regardless of whether a placebo or multi-ingredient supplement was ingested.

## Background

In 2016, it was estimated the dietary supplement industry has an economic impact of over $121 billion in the United States alone [1]. Approximately two-thirds of Americans report using dietary supplements, with half reporting regular consumption and nearly 20% utilizing sports supplements [2]. One popular category of sports supplements, termed “pre-workout” supplements, comprises products containing a mixture of biologically-active ingredients that purportedly enhance subjective feelings of energy, reduce fatigue and improve exercise performance. Despite the popularity of these products, a common frustration consumers and researchers encounter when evaluating pre-workout supplements is the frequent use of “proprietary blends,” which disguise the precise quantity of ingredients contained in the product. Proprietary blends on supplement facts labels contain a single value representing the cumulative mass of numerous ingredients without stating the quantity of each individual ingredient within the blend. Although most multi-ingredient pre-workout formulas available to consumers will never be directly examined in an experimental setting, the complete reporting of quantities of each individual ingredient allows consumers and health professionals to make more informed decisions regarding their usage. For example, there may be experimental evidence indicating an effective or optimal dose of a single ingredient, but if the quantity of the ingredient is masked in a proprietary blend, it is impossible to determine whether a scientifically-supported dose of the ingredient is reportedly present. Even when proprietary blends are not utilized, the multi-ingredient nature of most pre-workout supplements, as well as the different combinations and proportions of ingredients they contain, makes it difficult to determine which ingredients are responsible for observed performance effects [3].

Despite the limitations inherent to studying multi-ingredient supplements, the extreme prevalence of use and financial impact among active individuals and athletes necessitates formal evaluation of these products. Previous investigations of pre-workout supplements have revealed mixed results with respect to effects on muscular performance and subjective measures, although comparisons of previous studies may have limited utility due to differences in supplement formulations. Some supplements have improved subjective feelings of energy [4–6], while others have not [7, 8]. Likewise, there have been reports of improvements in resistance exercise performance, specifically in tests of repetitions-to-failure [4, 5, 7], while others found no benefits of pre-workouts relative to placebo for muscular endurance or strength during resistance exercise [6, 8–11]. These previous investigations have primarily utilized simple evaluations of performance, such as 1-repetition maximum tests and repetitions to failure during single or multiple sets. More nuanced assessments of resistance exercise performance (e.g. concentric and eccentric force production) over the course of a resistance exercise session are currently lacking.

Due to the limited information concerning the effects of pre-workout supplements on force production and subjective measures during resistance exercise, as well as the mixed results of previous investigations, it is important for researchers to continue to examine this category of dietary supplements. Therefore, the purpose of the present study was to examine the effects of two multi-ingredient pre-workout supplements on concentric and eccentric force production and subjective measures during a lower body resistance exercise session. It was hypothesized that, despite the differences in formulation, both supplements would increase force production and decrease subjective difficulty of the exercise session relative to placebo due to the presence of biologically-active ingredients that may enhance muscular performance.

## Methods

### Overview

**Fig. 1:**
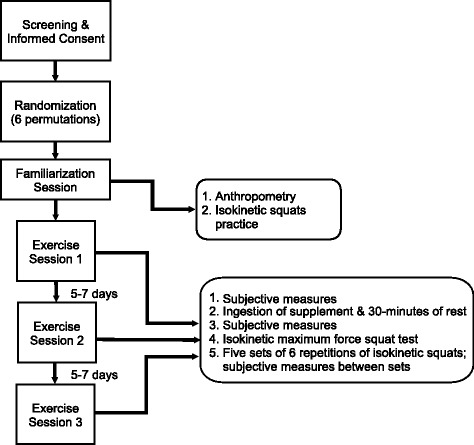
Study design

The present study was a counterbalanced, double-blind, placebo-controlled trial. Each participant completed one familiarization session followed by three identical exercise sessions (Fig. 1). At each exercise session, a different supplement was consumed: non-caffeinated pre-workout (NC), caffeinated pre-workout (C), or noncaloric placebo (P). The order of conditions was determined randomly and counterbalanced. The only investigator who was not blinded to the supplement conditions solely provided the dietary supplements to the participant, but played no role in data collection at exercise sessions. All investigators collecting data and all participants were blinded to the supplement conditions. Additionally, participants were informed that three different commercially-available dietary supplements were being tested, but were not informed that one of the supplements was a placebo.

### Participants

Resistance trained males and females were recruited for participation and screened for eligibility. While the inclusion criterion for training status was a minimum of 2 h per week of resistance training (RT) over the past 6 months, the actual RT experience of the sample was much greater (7.5 ± 3.9 h per week and 3.7 ± 2.6 years of RT experience). Participants were also required to be generally healthy (i.e. no known disease, medical condition, or orthopedic limitation that could affect exercise performance). Finally, only individuals who consumed a minimum of 100 mg of caffeine per day, as assessed by interview, were eligible to participate. All participants signed a consent form approved by the Texas Tech University Institutional Review Board prior to participation.

### Procedures

Prior to beginning exercise sessions, each participant completed a familiarization session. At the beginning of this visit, the details of the study procedures were explained to the participants, and the informed consent document was read and signed. Body weight and height were measured using a digital scale and stadiometer, and body fat percentage was assessed for descriptive purposes (Tanita BF-522 W). Participants were also trained how to complete the exercise tests they would subsequently perform at the exercise sessions, and each participant practiced executing these tests. Isokinetic squats were performed using a mechanized squat device (Exerbotics eSq, Tulsa, OK). Prior to performing squats, each participant’s preferred foot positioning was determined using a custom grid overlaid on the foot platform of the squat device. This foot positioning was recorded and utilized for all exercise sessions. Additionally, participants were asked to wear the same shoes at each visit to eliminate variability in force production due to footwear. No weight belts, knee wraps, or other such aids were allowed. During each squat, the participant placed his or her hands on the handles of the mechanized squat device and oriented the torso appropriately so that the designated pads rested on the upper trapezius. Prior to performing squats, each participant’s range of motion was determined. The range of motion was set to 90° between the thigh and lower leg at the bottom of the repetition and 170° at the top of the repetition. Each participant completed a maximum force production test, which consisted of 3 repetitions. For the first repetition, participants were instructed to give approximately 50% effort on the eccentric and concentric portions of the movement. For the second and third repetitions, participants were instructed to give maximal effort. Each of the repetitions consisted of a 4 s eccentric phase, followed by an approximately half-second pause at the 90° position and a 4 s concentric phase. After two minutes of rest, participants completed one set of 6 repetitions while again providing maximum effort. The cadence of the repetitions was identical to the maximum force production test (i.e. 4 s eccentric and concentric phases). This single set of 6 repetitions was identical to the sets used during the exercise sessions and was used to familiarize the participant with the mechanized squat device, although force was not recorded during the familiarization session.

After the familiarization session, each participant completed 3 exercise sessions, which were identical except for the pre-workout supplement consumed (NC, C, and P). Exercise sessions were separated by 5 to 7 days. The Random Sequence Generator, available at random.org, was utilized to randomly assign each participant to one of the 6 possible permutations of supplementation order (i.e. NC-C-P, NC-P-C, C-NC-P, C-P-NC, P-NC-C, P-C-NC). The study was counterbalanced so that an equal number of participants completed each possible supplementation order. Based on our sample size, each possible permutation was employed approximately four times (i.e. twice in male participants and twice in female participants). Each of the three exercise sessions was performed at the same time of day for a given participant, and all exercise sessions took place between 9:30 A.M. and 1:30 P.M. Participants were instructed to avoid lower body exercise for 72 h prior to each exercise session and to abstain from caffeine, alcohol and nicotine consumption overnight (≥12 h) prior to each session. Each participant was allowed to self-select his or her food intake prior to the exercise session. However, each participant was given a single day diet record and asked to record all energy intake prior to the exercise session on the day of testing. Participants were asked to eat as similarly as possible prior to each exercise session.

Two commercially available multi-ingredient pre-workout supplements were utilized in the present study (Carbon Prep [NC] and Jym® Pre-Jym [C]). Both supplements were purchased via online orders, and neither supplement manufacturer was involved in the study in any way. The supplement facts comparison is presented in Table 1. One noticeable difference between the supplements was the caffeine content: NC contained 0 mg of caffeine, whereas C contained 300 mg caffeine per serving. Both supplements and the placebo were similar in appearance and taste. Additionally, each beverage was provided to participants in a completely opaque cup with an opaque lid to prevent participants from observing the beverage. The commercially-available placebo beverage was chosen to mimic the taste of the pre-workout supplements without providing an appreciable amount of energy (5 kcal per serving). Male participants were given one full serving of each pre-workout supplement, and female participants were given 75% of one serving. This adjustment was made to account for the expectation of a smaller average body mass in female participants. Based on the average body weight of participants in this study, C provided 4.0 mg/kg of caffeine for males and 3.6 mg/kg of caffeine for females.

**Table 1:** Dietary supplement facts

	NC	C	P
Calories (kcal)	10	90	5
Carbohydrate (g)	3	3	–
Calcium (mg)	65	13	–
Citrulline Malate (g)	6	6	–
Creatine (g)	3^a^	2^b^	–
Betaine (g)	2.5	1.5	–
Alpha-Glyceryl Phosphoryl Choline (mg)	300	150^c^	–
Huperzine A (mcg)	200	50	–
Astragalus membranaceus root (g)	2	–	–
L-Carnitine L-Tartrate (g)	1	–	–
Cocoa seed powder (mg)	450	–	–
*Rhodiola rosea* root extract (mg)	180	–	–
Caffeine Anhydrous (mg)	–	300	–
Beta-Alanine (g)	–	2	–
Taurine (g)	–	1	–
N-Acetyl L-Cystine (mg)	–	600	–
*Beta vulgaris* L. (mg)	–	500	–
L-Leucine (g)	–	3	–
L-Isoleucine (g)	–	1.5	–
L-Valine (g)	–	1.5	–
L-Tyrosine (g)	–	1.5	–
BioPerine (mg)	–	5	–

^a^as monohydrate, ^b^as Hydrochloride, ^c^300 mg AlphaSize®, supplying 150 mg alpha-GPC

Prior to supplementation, the participant completed visual analog scales (VAS) rating his or her energy, fatigue, and focus. The VAS were administered via an iPad-based application (VasQ). VAS administered via iPad have previously been validated [12]. All VAS were grounded with phrases on the left and right sides of the line, but were unmarked between these phrases. The response of the participant was automatically converted to a score in millimeters, with 0 being the minimal score and 100 being the maximal score for each scale. The participant was then escorted to an adjacent laboratory space in which the pre-workout supplement was provided. Each supplement was provided in an opaque cup with an opaque lid, and the study investigator observed the participant consume the supplement within a 3-min period. After ingestion, the participant sat quietly for 30 min in the laboratory. Immediately after the 30-min waiting period, the VAS were repeated. The participant performed a self-selected bodyweight warm up of up to 5 min. The warm up activities were recorded by investigators, and the same warm up procedure was used at each trial. A 3-repetition maximum force production test identical to the test performed at the familiarization session was then performed. Due to the instruction to only provide 50% of maximal effort during the first repetition, the force data from the first repetition was discarded. Average concentric and eccentric peak force values were obtained using the output from the second and third repetitions. Following the maximum force production test, 5 sets of isokinetic squats were performed. Each set consisted of 6 repetitions, and 2 min of rest was allowed between sets. Participants were instructed to provide maximal effort on each repetition, and strong verbal encouragement was provided by blinded study investigators. The cadence of both the maximum force set and the exercise sets was identical to the familiarization sets, i.e. 4 s eccentric and concentric muscle actions with an approximately 0.5 s pause between muscle actions. Between each set, the VAS were repeated, and participants were instructed to provide their repetitions in reserve (RIR) rating, indicating how many additional repetitions they believe they could have completed. RIR was converted to a resistance exercise-specific rating of perceived exertion (RPE), as described by Zourdous et al. [13].

During the isokinetic squat exercise, the force signal was sampled from the load cell at 1 kHz (MP150WSW; Biopac Systems, Inc., Santa Barbara, CA), stored on a personal computer, and processed off-line using custom-written software (LabVIEW, Version 11.0; National Instruments, Austin, TX). The scaled force signal was low-pass filtered, with a 10-Hz cutoff (zero-phase lag, fourth-order Butterworth filter). All subsequent analyses were conducted on the scaled and filtered force signal. For each repetition of each set, isokinetic peak force was determined as the highest mean 25 ms epoch for both concentric and eccentric portions of the repetition. The single concentric and eccentric portions with the greatest peak force were designated as the maximal force values. Previous reliability statistics for these procedures from our laboratory have revealed intraclass correlation coefficients of 0.74 and 0.70 and standard error of measurement values expressed as a percentage of the mean of 8.8 and 10.6% for concentric and eccentric peak force data, respectively [14].

### Statistical analyses

An a priori power analysis indicated that 20 participants were needed to detect significant differences in maximal force production, based on an estimated effect size of .25, an α level of 0.05 and a power of 0.8. Data were checked for outliers via studentized residuals and visual inspection of boxplots for extreme cases. Most variables had no outliers. For variables containing outliers, the analyses were conducted both with and without extreme cases present. The results did not change appreciably based on the presence of outliers, so they were retained in the data. Normality of data was assessed via Shapiro-Wilk test and evaluation of skewness and kurtosis. Most subjective and force production variables were normally distributed, although several violations were present. However, due to the demonstrated robustness of ANOVA when normality is violated, data were not transformed for analysis [15]. Change in subjective variables and force production during exercise sets were analyzed using three-way repeated-measures ANOVA with supplement and time as within-subjects factors and sex as the between-subjects factors. Force production during the max test was analyzed by two-way repeated measures ANOVA with supplement as the within-subjects factor and sex as the between-subjects factor. Significant three-way interactions were followed up with simple two-way interactions using two-way repeated measures ANOVA. Significant simple two-way interactions were followed up with simple simple main effects using one-way repeated measures ANOVA. In the absence of a significant interaction, main effects were examined, and individual time points or supplements were evaluated using pairwise comparisons. Bonferroni post-hoc adjustments were utilized. Statistical significance was set at *p* < 0.05. Cohen’s d effect sizes were calculated for relevant dependent variables as the difference between means of NC or C and placebo, divided by the pooled standard deviation. Analyses were performed using IBM SPSS version 22.

## Results

After initial screening, 45 individuals were eligible for participation. Twenty-nine elected to participate, signed the informed consent document and began the study. Eight participants dropped out of the study (3 due to muscle strains during the exercise sessions, 3 due to failure to attend scheduled sessions, 1 due to injury outside the study, and 1 due to reported lack of time). Twenty-one participants (9 M, 12 F) completed all aspects of the study (Table 2). Of the 21 individuals who completed the study, 11 were Caucasian, 6 were Hispanic, 3 were Asian, and 1 was African American. There were no differences in dietary intake prior to each exercise session for calories (*p* = 0.67), carbohydrate (*p* = 0.81), fat (*p* = 0.65), protein (*p* = 0.72), sodium (*p* = 0.27), potassium (*p* = 0.99) or calcium (*p* = 0.66).

**Table 2:** Subject characteristics

	Females (*n* = 12)	Males (*n* = 9)	Combined (*n* = 21)
Age (y)	21.5 ± 2.0	20.7 ± 2.8	21.1 ± 2.4
Height (cm)	163.1 ± 6.4	175.0 ± 7.9	168.1 ± 9.1
Weight (kg)	63.2 ± 6.8	74.7 ± 14.0	68.2 ± 11.8
Body fat (%)	24.3 ± 7.2	10.2 ± 3.8	18.3 ± 9.2
RT experience (y)	3.6 ± 2.3	3.9 ± 3.0	3.7 ± 2.6
Weekly RT (h)	7.4 ± 3.7	7.7 ± 4.2	7.5 ± 3.9
Daily caffeine intake^a^ (mg)	271 ± 123	223 ± 106	250 ± 116

Mean ± SD

^a^67% of participants reported that pre-workout supplements were one of their regular sources of caffeine

**Fig. 2:**
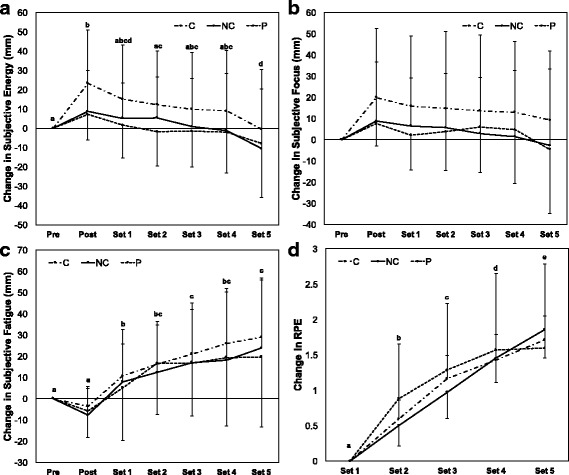
Subjective measures. **a** – **c**: Changes in subjective energy (**a**), focus (**b**) and fatigue (**c**) were assessed as the difference between visual analog scale scores between baseline and subsequent measurements. **d**: Ratings of perceived exertion were assessed after each exercise set using the repetitions-in-reserve method. Pre and post designations refer to pre-supplementation and post-supplementation assessments. A significant time main effect was present, and time points with different letter designations are significantly different from each other (*p* < 0.05)

No two- or three-way interactions were present for changes in VAS variables (*p* > 0.05 for all). Main effects for time were present for all subjective variables (*p* < 0.001 for energy; *p* = 0.004 for focus; p < 0.001 for fatigue; p < 0.001 for RPE). Feelings of energy and focus peaked after supplement ingestion and declined over the course of the exercise session, while subjective fatigue and RPE increased throughout the exercise session (Fig. 2). No condition or sex effects were present for these variables (p > 0.05 for all). Effect sizes for subjective measures, indicating the acute effects of dietary supplement ingestion, are displayed in Table 3.

**Table 3:** Effect sizes for acute effects of pre-workout supplements

	Energy				Focus				Fatigue			
	M		F		M		F		M		F	
	%	d	%	d	%	d	%	d	%	d	%	d
P	+8%	0.3	+17%	0.3	+8%	0.3	+17%	0.3	−16%	−0.1	−32%	−0.4
NC	+13%	0.5	+17%	0.3	+17%	0.5	+13%	0.3	−38%	−0.6	−7%	−0.1
C	+15%	0.7	+44%	0.8	+15%	0.5	+25%	0.4	−38%	−0.5	−20%	−0.5

Cohen’s d effect sizes calculated as the difference between pre- and post-supplementation means divided by the pooled standard deviation

There were no statistically significant interactions or supplement main effects for concentric or eccentric force production during the 3-repetition maximum test (concentric force: *p* = 0.46 for interaction, *p* = 0.31 for supplement; eccentric force: *p* = 0.39 for interaction, *p* = 0.57 for supplement). Although not statistically significant, average maximal concentric and eccentric forces in males were approximately 9% higher in the NC condition as compared to placebo (d = 0.3 relative to placebo) and approximately 5% higher in the C condition as compared to placebo (d = 0.1 to 0.2 relative to placebo). In females, average maximal concentric and eccentric force in the NC condition were virtually identical to placebo (0 to −1.5%; d = 0 to −0.1 relative to placebo), while the force appeared greater in the C condition (+4.5 to 11%; d = 0.2 to 0.4 relative to placebo). Sex main effects were present for concentric (*p* = 0.015) and eccentric force (*p* = 0.004), indicating that males produced greater force than females.

**Fig. 3:**
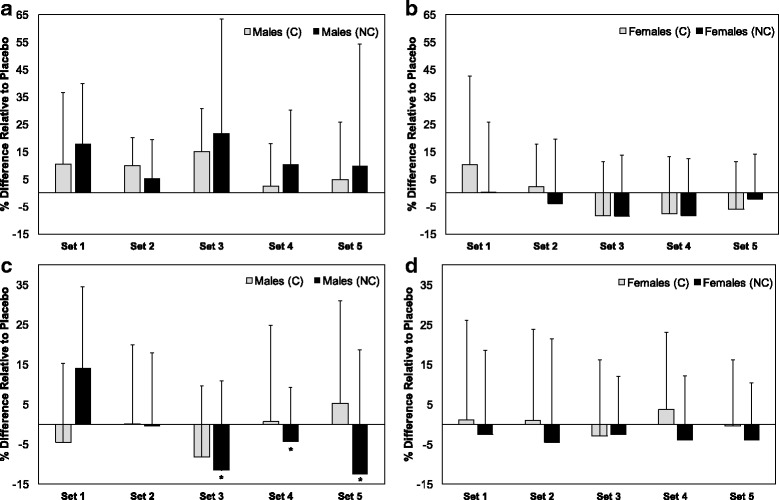
Effects of pre-workout supplements on force production. **a** – **b**: Maximal concentric force in males and females. **c** – **d**: Maximal eccentric force in males and females. Differences between the supplement conditions and placebo were calculated as the difference in means between conditions divided by the mean of the placebo condition. *significantly lower than set 1 (*p* < 0.05)

Concentric and eccentric force data from the 5 isokinetic exercise sets are presented in Table 4 and Fig. 3. Analysis revealed a three-way interaction for maximal eccentric force (Table 4). Follow up indicated a significant simple time*supplement interaction in males (*p* = 0.007), but not females (*p* = 0.95). A simple simple main effect for time was present in NC (*p* = 0.0001), but not C (*p* = 0.82) or P (*p* = 0.20). Pairwise comparisons revealed that maximal eccentric force production was lower in sets 3 (*p* = 0.026), 4 (*p* = 0.021), and 5 (*p* = 0.049) than set 1 in the NC condition. However, it should be noted that, in male participants, force production in set 1 was over 250 N greater on average in the NC condition as compared to the P and C conditions (*p* = 0.08), indicating that the statistically significant effects across the exercise sets may have been due to higher force production in set 1. Time main effects were present for concentric and eccentric forces, but this effect was only analyzed for concentric force due to the statistical interactions for eccentric force. For maximal concentric force, pairwise comparisons indicated that force production was lower in set 4 than set 1 (p = 0.02). No other differences between sets were statistically significant. Effect sizes for force production in the supplement conditions, relative to placebo, are presented in Table 5.

**Table 4:** Force production during exercise session

	Condition	Sex	Exercise Sets					*p* values for 3-way ANOVA with repeated measures						
			Set 1	Set 2	Set 3	Set 4^a^	Set 5	Supplement	Time	Sex	Supplement x Time	Supplement x Sex	Time x Sex	Supplement x Time x Sex
Maximal Concentric Force (N)	P	M	1449 ± 381	1395 ± 356	1328 ± 328	1321 ± 381	1466 ± 471	0.51	0.003^*^	0.26	0.31	0.08	0.17	0.53
		F	1303 ± 466	1312 ± 418	1407 ± 556	1342 ± 547	1313 ± 540							
	NC	M	1708 ± 463	1466 ± 333	1616 ± 601	1457 ± 380	1609 ± 459							
		F	1303 ± 345	1261 ± 318	1288 ± 482	1231 ± 338	1283 ± 376							
	C	M	1601 ± 488	1531 ± 419	1528 ± 381	1354 ± 360	1534 ± 468							
		F	1437 ± 409	1343 ± 374	1290 ± 354	1240 ± 276	1234 ± 328							
Maximal Eccentric Force (N)	P	M	1782 ± 563	1704 ± 591	1738 ± 724	1639 ± 707	1571 ± 613	0.63	0.001^*^	0.17	0.006^*^	0.93	0.005^*^	0.01^*^
		F	1428 ± 237	1412 ± 371	1469 ± 376	1366 ± 405	1414 ± 415							
	NC	M	2032 ± 590	1696 ± 679	1535 ± 551^*^	1567 ± 546^*^	1375 ± 562^*^							
		F	1391 ± 244	1348 ± 264	1431 ± 282	1312 ± 331	1358 ± 327							
	C	M	1700 ± 518	1706 ± 566	1594 ± 560	1651 ± 624	1652 ± 480							
		F	1444 ± 338	1427 ± 323	1425 ± 370	1415 ± 357	1408 ± 379							

^a^concentric force production during set 4 was lower than set 1 (p = 0.02)

^*^significantly different than other conditions, as indicated by significant pairwise comparisons (p < 0.05)

**Table 5:** Effect sizes for force production

		Maximal Concentric Force		Maximal Eccentric Force	
		M	F	M	F
Max Test	NC	0.27	0.00	0.24	−0.26
	C	0.42	0.42	0.08	0.09
Exercise Sets	NC	0.43	−0.14	−0.08	−0.15
	C	0.29	−0.06	−0.04	0.02

Cohen’s d effect sizes calculated as the difference between means of NC or C supplement and placebo, divided by the pooled standard deviation. The effect size for the exercise sets encompasses all five exercise sets

## Discussion

The present counterbalanced, double-blind, placebo-controlled trial examined the effects of two commercially available pre-workout supplements on concentric and eccentric force production during lower body resistance exercise. Both supplements contained 6 g of citrulline malate as the most prevalent ingredient. This compound has been reported to increase nitric oxide production [16, 17] and may decrease fatigue during exercise [18, 19], as well as increase training volume during resistance training [19]. Other ingredients with varying degrees of performance-enhancing potential (creatine [20], betaine [21, 22], alpha-glyceryl phosphoryl choline [23, 24] and huperzine A) were also present in both supplements. A number of specific ingredients were only present in one of the two supplements. Perhaps the most notable differences were the presence of 300 mg of caffeine and 6 g of branched chain amino acids per serving in the caffeinated supplement and the absence of these ingredients in the non-caffeinated supplement. Caffeine has been reported to increase power output and exercise volume at doses ≥3–5 mg/kg body weight [25–30], similar to the dose administered in the present study. However, there is uncertainty whether caffeine’s effects on resistance exercise performance are meaningful, as well as evidence that there may be “responders” and “non-responders” to caffeine intake [31]. The placebo in the present study contained only 5 kcal per serving and no active ingredients. Participants were not informed of the presence of a placebo condition and experienced similar increase in energy and focus and decrease in fatigue following acute supplement ingestion in all conditions. Despite this clear presence of the placebo effect and the lack of statistically different subjective responses between groups, effect size data indicate the possibility that the pre-workout supplements, particularly the caffeinated supplement, acutely enhanced some subjective measures to a slightly greater extent than the placebo. However, subjective responses during the exercise session were similar in all conditions: energy and focus were highest immediately after supplement or placebo ingestion and decreased progressively throughout the exercise session. Likewise, fatigue was lowest immediately post-ingestion, and fatigue and RPE increased progressively thereafter.

The incorporation of a 3-repetition maximal effort assessment prior to the exercise sets allowed for the examination of the effects of the supplements on maximal force production prior to fatiguing exercise. Despite the lack of statistically significant effects, examination of effect sizes and percent differences in force production, both of which were calculated relative to the placebo condition, allow for readily-interpretable metrics of possible supplement effects. These metrics revealed possible gender disparities between supplement conditions. Namely, force production in the NC condition was approximately 9% greater than placebo in males, but was 0 to 1.5% lower than placebo in females. In the C condition, force production in both males and females was 5 to 11% greater than placebo, although this was not statistically significant.

The results of the isokinetic exercise sets exhibited some similarities to the results of the max test, although differences were also apparent. Generally, significant differences in force production between conditions were not observed. In males, eccentric force production was lower during the later sets of the NC condition, as compared to the first set. This was not seen in the other conditions, although the apparently higher force production during set 1 in the NC condition likely influenced this result (i.e. high force production during the first set may have been at the expense of force production in later sets). In males, concentric force production was 5 to 20% higher during both supplement conditions as compared to placebo (d = 0.3 to 0.4 relative to placebo). However, in females, concentric force production appeared greater than placebo only during the first two sets in the C condition, and the overall effect of supplementation was minimal for both supplements (d = 0 to −0.1). In both sexes, neither supplement produced benefits relative to placebo in terms of eccentric force (d = −0.2 to +0.1).

It is important to emphasize that participants were not informed of the presence of a placebo condition in the present study. Additionally, due to the presence of three conditions, the counterbalanced and double-blind design, the similar appearance and presentation of the supplements and the similar subjective responses, we believe that participants remained unaware that one of the supplements was actually a placebo. Subsequently, since our effect sizes and percent differences for muscular performance were calculated relative to the placebo condition, they more closely reflect a true physiological effect than investigations in which participants are aware that they may be completing a placebo condition. Due to the strengths of study design, the present results may in fact underestimate the ergogenic effects of pre-workout supplements as they are typically utilized. An individual consuming a pre-workout supplement experiences not only any true physiological effects of the supplement, but also potentially powerful psychological effects. For individuals who believe in the effectiveness of pre-workout supplements, the alternative to consumption (i.e. abstaining from pre-workout ingestion) is unlikely to elicit the subjective increases in energy and decreases in fatigue elicited by the placebo in the present study. The real-world effect of a pre-workout supplement is the sum of psychological effects and any true physiological effects conferred by the active ingredients.

Due to the multi-ingredient nature of the pre-workout supplements employed in the present study, it is impossible to definitively state which compounds were responsible for the observed results. Additionally, very little is known about the specific interactions between different compounds contained in these supplements, despite some research support for the efficacy of individual ingredients. A consequence of these limitations is that results of separate investigations of pre-workout supplements may not be appropriately compared. While reports of improved exercise performance [4, 5, 7] or no benefits [6, 8–11] of pre-workouts have previously been presented, the differences in supplement formulations preclude appropriate comparisons.

Based on the nature of the mechanized squat machine used in the present study, eccentric and concentric force production should not be considered unrelated entities. Particularly due to the prolonged eccentric and concentric phases of each repetition (i.e. 4 s for each phase during each repetition), the effort expended in producing force during the concentric phase of a repetition likely had a direct impact on the ability to produce force during the eccentric phase of the subsequent repetition. Each set also affected subsequent sets such that increased force production in an earlier set potentially compromised force production in later sets. Additionally, although a resistance-trained population was utilized in the present study, none of the participants had prior experience with the mechanized squat device prior to familiarization. The device allowed for strict control of body positioning throughout each repetition, as well as the acquisition of a greater quantity of data than traditional strength testing. However, since the movement pattern of the device was fixed and the range of motion was prescribed by study investigators, the exercise should not be viewed as perfectly equivalent to a free-weight back squat, although it was designed to imitate one. In reality, each individual performs squats within the constraints of his or her anatomy and using his or her own unique combination of lifting form and cadence. Due to the controlled cadence of the device, as well as the inability of participants to alter the movement pattern, the effects of the dietary supplements on muscular performance during free-weight squats or other resistance exercises could potentially differ from the results observed in the present study.

## Conclusions

Due to the numerous available pre-workout supplements, each containing its own specific blend of ingredients in varying proportions, it is impossible to make definitive recommendations regarding their usage. In the present study, both pre-workouts contained 6 g of citrulline malate as the most prevalent ingredient, and a notable difference was that one supplement contained 300 mg of caffeine per serving, while the other was non-caffeinated. These dietary supplements did not definitively outperform the placebo, although there may have been a minor improvement in concentric force production in males. Importantly, the placebo effect may result in improved exercise performance due to modulation of feelings of energy and fatigue. Consequently, when resistance-trained subjects were unaware of the presence of a placebo, resistance exercise performance was similar regardless of whether a placebo or multi-ingredient supplement was ingested. The potentially powerful psychological influences, combined with possible physiological benefits of some specific formulations, may contribute to real-world effectiveness of certain pre-workout supplements.

## Abbreviations

ANOVA: Analysis of variance
C: Caffeinated pre-workout supplement
F: Female
M: Male
N: Newtons
NC: Non-caffeinated pre-workout supplement
P: Placebo
RIR: Repetitions in reserve
RPE: Rating of perceived exertion
RT: Resistance training
VAS: Visual analog scale
